# Hair follicle regeneration technology: from microenvironment regulation to functional reconstruction

**DOI:** 10.3389/fbioe.2026.1935385

**Published:** 2026-10-01

**Authors:** Shuxu Yang, Yunda Guo, Kui Ma, Siming Yang, Xiaobing Fu

**Affiliations:** 1 Research Center for Tissue Repair and Regeneration Affiliated to The Medical Innovation Research Department, PLA General Hospital and PLA Medical College, Beijing, China; 2 Fourth Medical Center of PLA General Hospital, Beijing, China

**Keywords:** alopecia, hair follicle regeneration, hair follicle microenvironment, microneedles, exosomes, hair follicle organoids

## Abstract

Hair loss is a common clinical condition that substantially impairs patients’ physical and mental health and quality of life. Hair loss is closely associated with disruption of hair follicle microenvironment homeostasis. Although current pharmacological treatments and hair transplantation can delay hair loss progression and improve local appearance, they cannot actively restore the damaged regenerative microenvironment of hair follicles. With advances in regenerative medicine, therapeutic strategies for hair loss increasingly focus on reconstructing hair follicles with regenerative capacity. Centering on the hair follicle microenvironment and functional reconstruction, this review first summarizes the hair follicle cycle and the microenvironmental and signaling mechanisms that regulate hair follicle stem cell quiescence and anagen entry. We then evaluate recent advances in drug delivery systems, regenerative therapies, and hair follicle tissue engineering. We analyze the progress and translational prospects of these strategies in modulating the hair follicle cycle and constructing engineered hair follicle organoids, aiming to achieve functional hair follicle reconstruction and scalable hair follicle production, thereby providing new insights for advancing hair loss treatment.

## 1 Introduction

Hair follicles are highly complex mini-organs in mammalian skin that continuously generate hair through cyclical self-renewal. This process depends on the precise coordination between hair follicle stem cells (HFSCs) and their surrounding mesenchymal niche, particularly the dermal papilla (DP) (Williams et al., 2020). Hair follicles are essential components of the skin barrier and participate in thermoregulation, sensory transmission, and social signaling. Their homeostasis is an important indicator of skin health.

Hair follicles possess regenerative potential, but their cyclical growth is susceptible to disruption by genetic predisposition, hormonal imbalances, immune microenvironment dysregulation, and physicochemical damage (Liang et al., 2023). These factors can lead to abnormalities in the hair follicle microenvironment and disruption of the hair follicle cycle, ultimately resulting in hair loss disorders. Androgenetic alopecia (AGA) is the most common type and is characterized by progressive hair follicle miniaturization under the influence of dihydrotestosterone, a shortened anagen phase, and eventual follicle regression (Liu et al., 2025). Hair loss affects physical appearance and frequently causes psychological issues such as anxiety and diminished self-confidence. Hair loss-related disorders are receiving increasing medical and social attention.

Current clinical treatments for hair loss primarily include pharmacological therapy and hair transplantation. Minoxidil and finasteride are first-line drugs approved by the U.S. Food and Drug Administration. Minoxidil prolongs the anagen phase through multiple mechanisms, including opening potassium channels, promoting angiogenesis, and activating the Wnt/β-catenin signaling pathway (Suchonwanit et al., 2019). Finasteride reduces dihydrotestosterone levels by inhibiting 5α-reductase, thereby delaying hair follicle miniaturization (Stout and Stumpf, 2010). However, pharmacological treatments show individual variability in efficacy, and relapse commonly occurs upon discontinuation. Finasteride is also associated with sexual function-related side effects. Hair transplantation can improve local hair density, but limited donor follicle availability, variable post-transplantation survival rates, and scar formation restrict its clinical application (Jimenez et al., 2021). For patients with severe depletion of donor hair or completely destroyed follicles, conventional therapeutic approaches have significant limitations.

Current efforts to treat hair loss increasingly integrate materials science, tissue engineering, and stem cell biology to move from improving the follicular microenvironment toward functional reconstruction. Delivery systems transport drugs or bioactive materials into the follicle and modulate regenerative signaling pathways for targeted intervention. Tissue-engineered follicles recapitulate follicular development and regulate the hair cycle during culture, enabling the resulting structures to acquire cyclical growth capacity. Although these strategies differ in their technical routes, they are generally organized around activating hair regeneration and improving the follicular microenvironment. This review focuses on drug delivery, regenerative therapies, and *in vitro* biofabrication. It compares their progress and clinical translation within a common framework centered on the hair follicle microenvironment and the shared signaling network that governs HFSC quiescence and anagen entry. Figure 1 provides an integrated overview of this framework, linking the hair follicle cycle, microenvironmental components, shared signaling pathways, therapeutic strategies, functional outcomes, and translational bottlenecks. Section 2 first outlines the hair follicle cycle and the mechanisms by which the microenvironment regulates hair follicle growth and development. The subsequent sections then evaluate advanced drug delivery systems, regenerative therapies, and tissue engineering strategies for functional hair follicle reconstruction.

**FIGURE 1:**
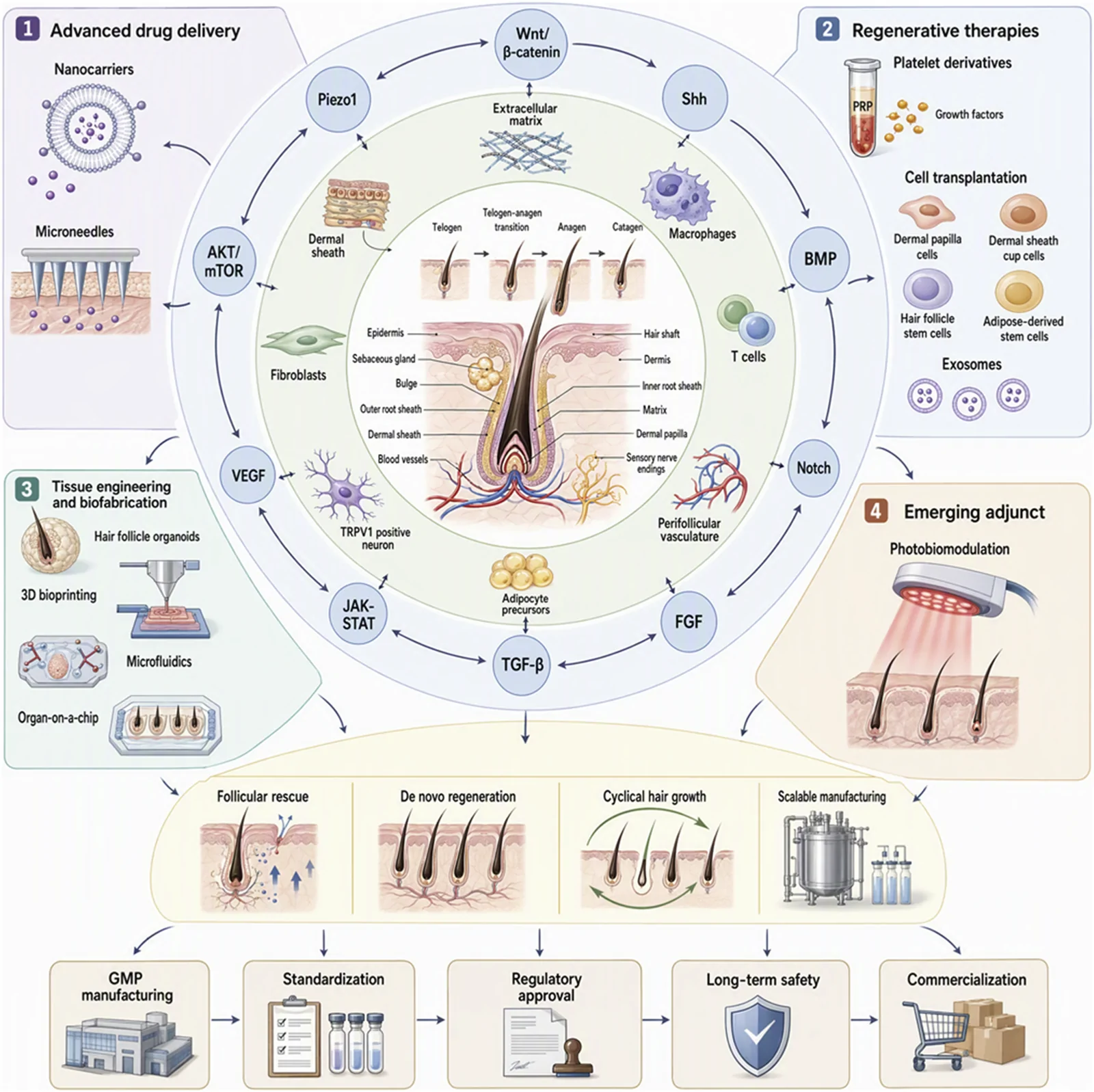
Overview of hair follicle regeneration from microenvironment regulation to functional reconstruction. The figure integrates the hair follicle cycle, microenvironmental components, shared signaling pathways, therapeutic strategies, functional outcomes, and translational bottlenecks. Abbreviations: GMP, good manufacturing practice; PRP, platelet-rich plasma; TRPV1, transient receptor potential vanilloid 1.

## 2 Hair follicle cycle and microenvironmental regulation mechanisms

Hair follicles are mini-organs in the skin that undergo cyclic regeneration (Zhang and Chen, 2024). After birth, hair follicles repeatedly pass through growth, regression, and resting phases throughout life (Sennett and Rendl, 2012). This process is driven by bidirectional signaling between hair follicle stem cells (HFSCs) and their surrounding microenvironment (Williams et al., 2020). The normal progression of the hair follicle cycle depends on the precise switching of HFSCs between quiescence and activation (Hu et al., 2021). It also requires the coordinated participation of multiple cell types, extracellular matrix (ECM), and signaling molecules (Correia et al., 2023). When these regulatory steps become abnormal, the hair follicle cycle is disrupted, leading to follicle miniaturization, a shortened anagen phase, and hair loss (Liang et al., 2023). Understanding the function of the hair follicle cycle, the mechanisms of cycle transition, and the influence of the microenvironment on hair follicle growth and development is essential for developing hair follicle regeneration strategies (Liu et al., 2025).

### 2.1 Functional features of the hair follicle cycle

The hair follicle cycle is divided into three main phases: anagen, catagen, and telogen (Schneider et al., 2009). Some studies also describe the transition from telogen to anagen as a separate phase (Natarelli et al., 2023). Hair follicles in different phases show distinct differences in structure, cell activity, and metabolic state (Rishikaysh et al., 2014).

Anagen is the most active phase of hair follicle growth (Schneider et al., 2009). During this phase, HFSCs are activated and migrate from the bulge to the hair bulb (Hu et al., 2021). They differentiate into transit-amplifying cells (TACs) (Hu et al., 2021). TACs continue to proliferate and differentiate upward to form the inner root sheath, outer root sheath, and hair shaft (Millar, 2002). The dermal papilla (DP) is located at the base of the hair bulb (Sennett and Rendl, 2012). Through signaling exchange with matrix cells and HFSCs, the DP maintains the duration of anagen (Genander et al., 2014). During anagen, the DP volume increases, angiogenesis increases, and perifollicular blood supply becomes abundant (Yano et al., 2001). In the human scalp, approximately 85%–90% of hair follicles are in anagen, and this phase can last from two to 7 years (Natarelli et al., 2023).

Catagen is the transition from growth to quiescence (Schneider et al., 2009). Matrix cells and outer root sheath epithelial cells undergo apoptosis (Foitzik et al., 2000). The lower part of the hair follicle rapidly regresses (Millar, 2002). The DP volume shrinks and migrates upward along the epithelial strand (Heitman et al., 2020). It finally localizes below the bulge (Martino et al., 2021). Melanocytes stop producing melanin, and hair shaft growth stops (Rishikaysh et al., 2014). Catagen is relatively short, lasting about two to 4 weeks (Natarelli et al., 2023). Only 1%–2% of hair follicles in the human scalp are in this phase (Natarelli et al., 2023). The upward migration of the DP is a key step for normal hair follicle cycle switching (Heitman et al., 2020). If the DP fails to migrate below the bulge, the hair follicle cannot enter the next anagen phase and eventually is lost (Martino et al., 2021).

Telogen is the relatively quiescent phase (Schneider et al., 2009). HFSCs remain in the bulge and stay quiescent (Hu et al., 2021). The DP is located near the bulge and releases inhibitory signals to maintain HFSC quiescence (Genander et al., 2014). The hair shaft forms a club hair and eventually sheds (Millar, 2002). In the human scalp, about 10%–15% of hair follicles are in telogen, which lasts about 3 months (Natarelli et al., 2023). At the end of telogen, the DP releases activation signals (Plikus et al., 2008). HFSCs are reactivated, and the hair follicle enters the next anagen phase (Hsu et al., 2011).

### 2.2 Molecular mechanisms of hair follicle cycle transition

Hair follicle cycle transition depends on the activity changes of multiple signaling pathways at different phases (Rishikaysh et al., 2014). These pathways show different expression states during telogen, the telogen-to-anagen transition, anagen, and catagen (Hu et al., 2021). They regulate HFSC quiescence and activation through mutual inhibition or maintenance (Genander et al., 2014).

During telogen, HFSCs are located in the bulge and remain quiescent (Plikus et al., 2008). BMP2 and BMP4 are highly expressed in the DP (Genander et al., 2014). BMP signaling inhibits HFSC activation through the Smad1/5/8 pathway (Genander et al., 2014). Wnt signaling has low activity at this stage, and β-catenin is difficult to stabilize (Choi, 2020). BMP signaling maintains HFSC quiescence and prevents premature entry into anagen (Wang et al., 2022).

During the telogen-to-anagen transition, Noggin and TGF-β2 expression increases in the DP (Oshimori and Fuchs, 2012). Noggin inhibits BMP signaling and removes the inhibition of HFSCs (Plikus et al., 2008). At the same time, Wnt ligand expression increases in the hair germ (Myung et al., 2013). Wnt binds to Frizzled receptors and LRP co-receptors, inhibiting β-catenin phosphorylation (Choi, 2020). Stabilized β-catenin enters the nucleus and binds to LEF/TCF transcription factors (Hu et al., 2021). This activates downstream genes such as AXIN2, LEF1, and LGR5 (Wen et al., 2025). HFSCs are activated and migrate from the bulge to the hair bulb (Hsu et al., 2011). Shh (Sonic Hedgehog, Shh) is then expressed in the hair germ (Woo et al., 2012). Through Patched and Smoothened receptors, Shh promotes downward migration of the DP and maintains Wnt signaling activity (St-Jacques et al., 1998). TGF-β2 also participates in HFSC activation (Hibino and Nishiyama, 2004). These signals together drive the hair follicle into anagen (Rishikaysh et al., 2014).

During anagen, Wnt, Shh, and Notch signaling together support matrix cell proliferation and hair shaft differentiation (Schneider et al., 2009). Wnt signaling maintains the proliferative state of HFSCs (Choi, 2020). Shh signaling promotes DP maturation and perifollicular angiogenesis (Woo et al., 2012). Notch signaling regulates the balance between HFSC proliferation and differentiation through the Dll1 ligand (Yamamoto et al., 2003). FGF7 and FGF10 participate in HFSC maintenance (Hébert et al., 1994). VEGF-A binds to VEGFR1/2 and promotes endothelial cell proliferation and migration, increasing perifollicular blood supply (Yano et al., 2001). DP cells secrete EDN1, which acts on endothelial cells to promote angiogenesis (Bassino et al., 2015). Endothelial cells also secrete factors that support DP cell function (Zhou et al., 2024).

During catagen, TGF-β1 expression increases and induces apoptosis of matrix cells and outer root sheath epithelial cells (Foitzik et al., 2000). FGF5 and FGF18 drive the transition from anagen to catagen (Hébert et al., 1994; Carrion et al., 2024). Wnt antagonist expression increases and inhibits Wnt signaling (Choi, 2020). The DP volume shrinks and migrates upward along the epithelial strand (Heitman et al., 2020). Melanocytes stop producing melanin, and hair shaft growth stops (Rishikaysh et al., 2014). After catagen, the hair follicle enters telogen (Schneider et al., 2009). BMP signaling increases again and maintains HFSC quiescence (Genander et al., 2014). The DP localizes below the bulge and prepares for the next activation cycle (Plikus et al., 2008)

In alopecia areata, CD8+NKG2D+ T cells attack hair follicles through IFN-γ/JAK-STAT signaling (Xing et al., 2014). This disrupts hair follicle immune privilege and leads to hair follicle structural destruction and hair loss (Xing et al., 2014). JAK inhibitors can block this signaling and restore hair growth (King et al., 2022).

Together, these pathways form the regulatory network that governs HFSC quiescence and activation, and they are summarized in Table 1.

**TABLE 1:** Core signaling pathways regulating hair follicle regeneration and their therapeutic implications.

Signaling pathway	Role in hair follicle cycle regulation	Key ligands and downstream effectors	Primary target cell types	Aberrant activity in alopecia	References
Wnt/β-catenin	Master switch for anagen entry: activates HFSCs from quiescence to proliferation maintains the hair-inducing capacity of DPCs	Wnt3a, Wnt10b β-catenin, LEF1/TCF GSK-3β (negative regulator)	HFSCs (bulge) DPCs Hair matrix cells	Attenuated Wnt signaling in AGA leads to shortened anagen and follicular miniaturization	(Wen et al., 2025; Oak et al., 2025)
BMP	Gatekeeper of HFSC quiescence: nuclear pSMAD1/5 is elevated in quiescent HFSCs during telogen and must decline to permit anagen entry	BMP2/4/6 SMAD1/5/8→SMAD4 Noggin (endogenous antagonist)	HFSCs DPCs TAC progenitor cells	Persistent BMP hyperactivity prevents HFSC activation prolongs telogen, and impairs hair regeneration	(Genander et al., 2014; Ge et al., 2019)
Shh/Hedgehog	Driver of hair germ downgrowth and proliferation: the Notch effector Hes1 modulates Shh responsiveness to initiate anagen	Shh Gli1/2 (transcription factors) Ptch1/Smo Hes1	Hair matrix cells ORS keratinocytes Secondary hair germ (HG)	Attenuated Shh signaling is associated with follicular miniaturization and reduced hair shaft diameter	(Suen et al., 2020)
Notch	Modulates HFSC proliferation-differentiation balance miR-1285-3P targets the NOTCH pathway to regulate HFSC proliferation and differentiation	Notch1–4 Jagged/Delta ligands Hes1 (downstream effector)	HFSCs Keratinocytes HF epithelial lineages	NOTCH pathway dysregulation is associated with disrupted HFSC proliferation-differentiation homeostasis	(Demehri and Kopan, 2009; Yan et al., 2023)
FGF	Governs hair cycle duration: FGF5 is a key driver of the anagen-to-catagen transition FGF5/FGF18 suppression promotes hair growth	FGF5, FGF18 FGFR1ERK/MAPK pathway	DPCs ORS keratinocytes Dermal fibroblasts	Loss-of-function FGF5 mutations produce the angora phenotype (abnormally long hair) in mice	(Hébert et al., 1994; Zhao et al., 2021)
TGF-β	Pleiotropic regulator of the hair cycle: TGF-β1 induces catagen entry *in vivo* TGF-β2 promotes HFSC activation during the telogen–anagen transition ASC-derived exosomal miRNAs target TGF-β1/SMAD2 to treat AGA	TGF-β1, TGF-β2 SMAD2/3 miR-574-3p, miR-125a-5p	DPCs Hair matrix cells ORS keratinocytes HFSCs	Hyperactivation of TGF-β1/SMAD2 signaling is implicated in AGA-associated follicular miniaturization	(Oshimori and Fuchs, 2012; Myung et al., 2013; Wen et al., 2025)
JAK-STAT	Immune–HF interface: CD8+NKG2D+ T cells drive AA through IFN-γ/JAK-STAT signaling, disrupting HF immune privilege JAK inhibitors reverse disease	JAK1–3 STAT1/3/5 IFN-γ, IL-15 NKG2D	HFSCs (immune-privileged niche) CD8+NKG2D+ T cells DPCs	Hyperactivation of JAK-STAT signaling in AA disrupts HF immune privilege, leading to T-cell-mediated HF destruction	(Xing et al., 2014; King et al., 2022)
VEGF	Pro-angiogenic support: restores perifollicular microvasculature to supply nutrients and oxygen essential for hair growth	VEGF-A VEGFR1/2 HIF-1α	DPCs Vascular endothelial cells ORS keratinocytes	Insufficient perifollicular vascularization in the bald scalp of AGA patients contributes to follicular miniaturization	(Ding et al., 2024)

### 2.3 Composition and function of the hair follicle microenvironment

The hair follicle microenvironment is a complex three-dimensional structure surrounding the hair follicle (Correia et al., 2023). It is composed of multiple cell types, ECM, and signaling molecules (Correia et al., 2023). The microenvironment provides structural support for HFSCs (Jiang et al., 2026). It also regulates HFSC quiescence, activation, and differentiation through secreted factors and mechanical signals (Lei et al., 2023). Microenvironment components include ECM, immune cells, vascular endothelial cells, adipocyte precursors, nerve endings, fibroblasts, and the dermal sheath (Bensa et al., 2023).

ECM is an important component of the microenvironment (Jiang et al., 2026). Collagen networks continuously remodel around the hair follicle (Jiang et al., 2026). Their organization and mechanical state influence whether HFSCs enter proliferation (Jiang et al., 2026). Collagen fibers interact with HFSCs through integrin receptors and transmit mechanical signals (Lei et al., 2023). Matrix metalloproteinases participate in ECM degradation and remodeling, and their expression changes dynamically during hair follicle cycle transition (Buffoli et al., 2014). Increased ECM stiffness can promote HFSC activation (Lei et al., 2023). Excessive fibrosis is associated with irreversible hair follicle destruction in scarring alopecia (Halley-Stott et al., 2020). Changes in ECM stiffness can affect integrin signaling and thereby alter HFSC proliferation (Lei et al., 2023).

Immune cells play a key role in maintaining hair follicle immune privilege (Christoph et al., 2000). Regulatory T cells and macrophages are present around the bulge and hair bulb (Cohen et al., 2024). Regulatory T cells maintain hair follicle immune tolerance by secreting anti-inflammatory factors such as IL-10 (Cohen et al., 2024). Macrophages participate in HFSC activation during the telogen-to-anagen transition (Castellana et al., 2014). In alopecia areata, CD8+NKG2D+ T cells attack hair follicles, disrupt immune privilege, and cause hair follicle inflammation and hair loss (Xing et al., 2014). Immune microenvironment dysregulation is a core mechanism in the pathogenesis of alopecia areata (King et al., 2022). IL-10 secreted by regulatory T cells can suppress perifollicular inflammation and maintain hair follicle immune tolerance (Cohen et al., 2024).

Vascular endothelial cells have bidirectional signaling exchange with DP cells (Zhou et al., 2024). Endothelial cells secrete EDN1, which activates taurine metabolism in DP cells and promotes hair growth (Zhou et al., 2024). DP cells release CTF1, which activates α-linolenic acid metabolism in endothelial cells and promotes angiogenesis (Zhou et al., 2024). This bidirectional signaling weakens during aging and may be an important mechanism of senile alopecia (Zhou et al., 2024). Perifollicular vascular degeneration is associated with follicle miniaturization in androgenetic alopecia (Yano et al., 2001). The EDN1 and CTF1 signaling exchange between endothelial cells and DP cells participates in hair follicle cycle regulation (Bassino et al., 2015).

Adipocyte precursors participate in hair follicle cycle regulation (Festa et al., 2011). Adipocyte precursors secrete factors such as PDGF, which act on HFSCs and affect their activation (Festa et al., 2011). During the hair follicle cycle, adipocyte precursors proliferate during anagen and decrease during catagen (Festa et al., 2011). These changes are synchronized with the hair follicle cycle (Festa et al., 2011).

Nerve endings are closely associated with hair follicles (Ben-Shaanan et al., 2024). Sensory nerve endings are present around hair follicles and express receptors such as TRPV1 (Bodó et al., 2005). Nerve endings release transmitters such as calcitonin gene-related peptide, which can affect HFSC activity (Ben-Shaanan et al., 2024). Shh signaling is expressed in nerve endings and participates in hair follicle cycle transition (Zhou et al., 2024).

Fibroblasts and the dermal sheath constitute the mesenchymal microenvironment of the hair follicle (Martino et al., 2021). The dermal sheath wraps the outer layer of the hair follicle and is homologous to DP cells (Martino et al., 2021). Dermal sheath cells participate in DP migration and hair follicle regeneration during the hair follicle cycle (Heitman et al., 2020). Fibroblasts show functional heterogeneity, and different subpopulations have different roles in matrix synthesis and hair follicle induction (Bensa et al., 2023). Cytokines and growth factors secreted by fibroblasts can affect HFSC and DP cell function (Bensa et al., 2023).

In androgenetic alopecia, the microenvironment undergoes multiple changes (Liu et al., 2025). DP cell volume shrinks, function declines, and expression of senescence markers such as p16INK4a increases (Bahta et al., 2008). DP cell secretion of Wnt, BMP, FGF, and Shh signaling factors decreases, while inhibitory factors such as TGF-β, DKK-1, and IL-6 increase (Upton et al., 2015). ECM remodeling becomes abnormal, and collagen network organization is disrupted (Jiang et al., 2026). Perifollicular blood vessels decrease, and the immune microenvironment changes (Yano et al., 2001). These changes together lead to follicle miniaturization and a shortened anagen phase (Liu et al., 2025).

In scarring alopecia, hair follicles are completely destroyed (Halley-Stott et al., 2020). Hair follicle structures disappear and are replaced by fibrous tissue (Larrondo and McMichael, 2023). HFSCs and DP cells are absent, and the microenvironment structure is irreversibly damaged (Halley-Stott et al., 2020). In this situation, drug and cell therapies are difficult to restore hair follicle regeneration, and reconstruction of hair follicle structure is required (Larrondo and McMichael, 2023).

The hair follicle microenvironment is a key regulatory factor for hair follicle regeneration (Correia et al., 2023). Understanding the composition and function of the microenvironment, as well as the role of microenvironment components in the hair follicle cycle, can help develop therapeutic strategies targeting the microenvironment (Jiang et al., 2026). Drug delivery systems, regenerative therapies, and tissue engineering strategies can all promote hair follicle regeneration by modulating the microenvironment (Correia et al., 2023; Bensa et al., 2023).

## 3 Advanced drug delivery strategies

Conventional hair loss pharmacotherapies suffer from multiple limitations in drug delivery. Although first-line drugs such as minoxidil and finasteride have been approved by the FDA, topical formulations are hindered by the stratum corneum barrier, resulting in low transdermal absorption and limited drug accumulation at the hair follicle site (Weichert et al., 2025). Oral administration, in turn, carries the risk of systemic side effects. These limitations fundamentally arise from the reliance of conventional formulations on passive diffusion for drug distribution, lacking the capacity for targeted and precise delivery to hair follicles.

Advanced drug delivery strategies, such as nanocarriers and microneedles, present new opportunities to address the above challenges. The selective enrichment of nanomaterials in hair follicles and the physical penetration of microneedles can overcome the stratum corneum barrier, substantially improving transdermal drug permeation efficiency. Furthermore, through particle size optimization, surface modification, or charge effects of carrier materials, the follicular targeting specificity of drugs can be further enhanced, enabling sustained and controlled release within hair follicles to maintain effective local concentrations.

This section introduces the advantages of drug delivery technologies such as nanocarriers and microneedles in the treatment of hair loss, with a focus on recent technological advances and clinical translational challenges.

### 3.1 Nanoscale delivery systems

Nanoscale delivery systems represent one of the most extensively investigated strategies for follicle-targeted delivery. The hair follicle openings and their surrounding lipid-rich environment provide natural entry channels for nanoparticles, preferentially enriching nanocarriers with particle sizes in the range of 200–500 nm (Patzelt and Lademann, 2013). Compared with free drugs, encapsulating drugs within nanocarriers can significantly increase their accumulation at the hair follicle site, prolong local retention time, and simultaneously reduce the amount of drug entering the systemic circulation via the transdermal route, thereby lowering the risk of systemic side effects. Nanoscale delivery systems currently under investigation primarily include lipid-based nanocarriers, polymer-based nanocarriers, drug nanocrystals, and composite multifunctional nanoplatforms.

Lipid-based carriers include liposomes, nanostructured lipid carriers (NLCs), and others. Their lipid components possess natural compatibility with sebum on the skin surface, enabling accumulation in hair follicles via the lipid channels of the pilosebaceous unit. For instance, protopanaxadiol liposomes loaded with resveratrol (PPD-Lip@RES) can traverse the stratum corneum and achieve therapeutic concentrations around hair follicles (Zhang X. et al., 2024). Nanostructured lipid carriers constructed with squalene as the matrix (squarticles) selectively target hair follicles by exploiting the interaction between squalene and physiological sebum; these lipid carriers have an average particle size of 229 nm, a drug encapsulation efficiency of 82.72%, and approximately 11% of the drug can be deposited in hair follicles (Fereig et al., 2026).

Polymer nanoparticles are mainly constructed from natural biodegradable polymers and synthetic biodegradable polymers, possessing favorable drug-loading and sustained-release capabilities. The former include chitosan, sodium alginate, hyaluronic acid, and gelatin, while the latter include poly (lactic-co-glycolic acid) (PLGA), polycaprolactone (PCL), and poly (lactic acid) (PLA). Among natural biodegradable polymers, chitosan is one of the most extensively studied, owing to its excellent biocompatibility and its capacity to facilitate drug skin penetration (Guzmán et al., 2022). For example, chitosan-coated minoxidil nanocapsules (ChiNCs) exhibit a drug-loading capacity as high as 50 wt%, favorable controlled-release properties, and colloidal stability, with skin penetration ability significantly superior to that of the free drug (Lee et al., 2026). In addition, chitosan itself possesses hair growth-promoting activity (Azuma et al., 2019). Investigators have loaded five drugs, including phenobarbital, separately into chitosan nanoparticles and applied them in a cyclophosphamide-induced alopecia mouse model; the results showed that all five drugs alleviated hair dystrophy, improved hair quality, and effectively prevented chemotherapy-induced alopecia (Fereig et al., 2025). PLGA, as a synthetic biodegradable polymer, likewise possesses excellent biocompatibility (Makadia and Siegel, 2011). PLGA allows precise control of its degradation time by adjusting the ratio of lactic acid to glycolic acid; owing to its gradient-degradable property, it has been employed to construct long-acting sustained-release microspheres capable of achieving continuous drug release (Yin et al., 2023; Shin et al., 2025).

Drug nanocrystals (NCs) refer to nanoscale crystalline particles composed of pure active pharmaceutical ingredients (APIs) with sizes less than 1,000 nm (Alnaim, 2024). Their defining characteristic lies in their ability to markedly increase the apparent solubility, dissolution rate, and bioavailability of poorly soluble drugs (Sharma et al., 2026; Alkhaldi and Keck, 2025). Existing studies have applied nanocrystals to the intervention of alopecia areata and androgenetic alopecia, exploring their potential to mediate highly efficient targeted drug delivery to hair follicles. For example, one study formulated the corticosteroid drug clobetasol-17-propionate into a nanocrystal gel (CP-NC-Gel). Compared with conventional clobetasol gel, CP-NC-Gel achieved a fourfold increase in follicular drug deposition in *ex vivo* skin permeation experiments, while its deposition in the stratum corneum and dermis was also significantly higher, enabling sustained local release with reduced systemic exposure (Kumar et al., 2025). For androgenetic alopecia, researchers have developed quercetin nanocrystals, baicalin nanocrystals (BA-NCs), chitosan-coated finasteride nanocrystals (CS-FIN-NCs), and others (Su et al., 2025; Zhao et al., 2025a; Xiong et al., 2026). Combining nanocrystals with microneedle delivery technology can further enhance drug accumulation in hair follicles and construct long-acting sustained-release systems. This strategy can effectively reduce dosing frequency, thereby improving patient compliance with long-term treatment.

Notably, certain nanomaterials themselves possess bioactive functions, capable of modulating signaling pathways related to the hair cycle and actively regulating the hair follicle microenvironment. Materials that have been experimentally validated include polydopamine nanoparticles, conjugated linoleic acid (CLA), cerium oxide nanoparticles (CeNPs), hyperbranched polymer dots (HPD), and others (Chang CC. et al., 2025; Yang et al., 2024; Dong et al., 2025; Lan L. et al., 2026; Zhang et al., 2022). For example, HPD, as a hyperbranched polyamic acid nanomaterial, combines nanozyme antioxidant activity with high transdermal penetration capacity, enabling activation of the Wnt/β-catenin pathway, promotion of HFSC proliferation, and simultaneous scavenging of free radicals in the hair follicle microenvironment to reduce follicle aging damage (Chang CC. et al., 2025). Building on these advances, nanoscale delivery systems are progressively evolving from simple drug encapsulation toward integrated multifunctional platforms. These platforms incorporate innovations in material composition, structural design, and drug loading to construct nanocarriers with higher drug-loading capacity, enhanced transdermal efficacy, and the concurrent ability to improve the microenvironment and modulate hair cycle-related pathways (Thepphankulngarm et al., 2024; Sun G. et al., 2025). For instance, Chen et al., based on the “seed-and-soil” theory of hair follicles, constructed TFC multifunctional nanoparticles through metal-coordination self-assembly of curcumin, tannic acid, and Fe^3+^. Among these components, curcumin inhibits the Akt/mTOR pathway to activate hair follicle cell autophagy, while the nanocarrier constructed via tannic acid-iron coordination assembly possesses potent reactive oxygen species-scavenging capacity and enzyme-like activity, capable of ameliorating the oxidative stress-damaged hair follicle microenvironment. In an AGA mouse model, TFC combined with microneedle delivery accelerated hair growth, demonstrating superior efficacy to minoxidil with fewer side effects (Chen et al., 2025a).

In summary, nanoscale delivery technologies are advancing from simple drug loading toward multifunctional delivery systems with active regulatory capabilities. By optimizing nanocarrier parameters such as particle size and surface modification, their delivery efficiency, stability, and follicle-targeting specificity can be further enhanced (Heydari et al., 2024; Atrooz et al., 2024). However, the majority of current research on nanoscale delivery technologies still relies on rodent models; more clinically relevant data need to be obtained using human hair follicle organoids and *ex vivo* scalp models in the future. The batch-to-batch reproducibility, long-term safety, and scalable manufacturing of nanoscale delivery systems remain challenges to be addressed for their clinical translation.

### 3.2 Microneedles

In recent years, microneedle technology has emerged as a research hotspot in the field of transdermal drug delivery for hair loss treatment. Microneedle arrays consist of hundreds of micron-scale needle tips (typically 100–1,000 μm in height) that can penetrate the stratum corneum barrier and form microchannels to substantially improve drug delivery efficiency. Meanwhile, the mechanical stimulation generated by microneedles can itself promote hair follicle entry into the anagen phase by activating the Wnt/β-catenin signaling pathway (Hong et al., 2025). Based on differences in drug release mechanisms, microneedles can be categorized into solid microneedles, hollow microneedles, coated microneedles, dissolving microneedles, and hydrogel microneedles. Among these, dissolving microneedles (DMNs) are the most extensively investigated type in preclinical studies for hair loss treatment, owing to their ease of fabrication, favorable biocompatibility, and the absence of biohazardous sharp waste after use.

A large body of preclinical research has validated the effectiveness of microneedles in delivering various active agents, including synthetic and natural hair growth-promoting drugs, nanomedicines, biologics, stem cells, and their derivatives. Microneedles significantly enhance the transdermal efficiency and follicular targeting of conventional drugs such as minoxidil, finasteride, and dutasteride; for example, strategies such as forming micelles or nanocrystals, incorporating cationic materials, and integrating PLGA microspheres have achieved substantially increased follicular drug accumulation and long-term sustained drug release (Yin et al., 2023; Zhao et al., 2025a; Zhang et al., 2026). Plant extracts contain a variety of bioactive components capable of targeting different pathogenic pathways. However, such components generally have poor aqueous solubility and low bioavailability. Microneedles combined with nanocarriers can effectively address these issues (Xiong et al., 2026). For example, a gas-propelled microneedle patch loaded with quercetin/puerarin nanoparticles (PQFN) generates bubbles through the reaction between foaming agents and interstitial fluid to drive deep drug diffusion, significantly increasing intrafollicular drug accumulation and achieving hair follicle regeneration through ROS scavenging, promotion of angiogenesis, and inhibition of DPC senescence (Chen et al., 2025b). Other representative agents include Platycladus orientalis leaf extract microneedles and stevioside-based microneedles, which synergistically promote hair growth through multiple mechanisms such as antioxidant, anti-inflammatory, and pro-angiogenic effects (Hong et al., 2025; Zhang et al., 2026).

Protein- or nucleic acid-based macromolecules, such as recombinant type XVII collagen, recombinant filaggrin-2, HDAC1-overexpressing extracellular vesicles, and IGF-1, can all be delivered transdermally via microneedle platforms, activating pathways including Wnt/β-catenin, MAPK/Erk, and VEGF/VEGFR to promote hair follicle regeneration (Ye et al., 2025; Suo et al., 2025; Sun et al., 2026; Chang et al., 2026; Xing et al., 2025a; Xing et al., 2025b). Microneedles combined with nanomedicine delivery can both increase the delivery efficiency of nanomedicines and achieve long-term drug release (Zhang S. et al., 2024). For instance, platinum nanozyme microneedles can scavenge ROS and restore oxidative phosphorylation in HFSCs. Compared with daily minoxidil administration, these microneedles require dosing only once every 3 days while yielding superior hair regeneration outcomes (Hu et al., 2024).

For stem cells and their derivatives, microneedles also demonstrate unique advantages. Chitosan lactate/adipose stem cell exosome separable microneedles, through sustained release of exosomes and L-lactic acid, promote DPC proliferation and regulate the hair follicle cycle, exhibiting hair regeneration effects superior to minoxidil within 7 days (Shi et al., 2022). More strikingly, the matrix-free hair follicle organoid delivery technology based on cryomicroneedle array patches (cryoMAP) circumvents the inhibition of organoid growth caused by hydrogels. Following transplantation, the organoids achieved hair-penetrating skin regeneration within 15 days, with a success rate exceeding 86%, opening a new avenue for hair follicle organoid transplantation in regenerative medicine (Wang S. et al., 2026).

Clinical studies employing microneedles as monotherapy or in combination with other treatments have been conducted in the field of hair loss treatment, all yielding encouraging evidence. For androgenetic alopecia, the efficacy of microneedles combined with minoxidil or finasteride has been preliminarily validated. A randomized controlled study enrolling 45 male AGA patients divided subjects into three groups: topical 5% minoxidil alone (Group A), minoxidil plus oral finasteride (Group B), and microneedles combined with 5% minoxidil plus finasteride (Group C). After treatment, patients in Group C demonstrated superior improvements in hair density, hair shaft diameter, and Norwood-Hamilton classification compared with the drug-only groups (Chang Y. et al., 2025). Clinical trials using microneedles as an adjunct to deliver other drugs have also been successively conducted; for instance, microneedles combined with 1% cetirizine, 2% chitosan, or dutasteride have all reported positive hair regrowth-promoting effects (Gohary et al., 2025; Wan et al., 2026). Notably, in a study on dutasteride microneedle treatment, the placebo control group receiving microneedle puncture alone also exhibited a certain degree of efficacy, providing clinical evidence for the therapeutic value of mechanical stimulation itself independent of pharmacological agents (Gasques et al., 2025). In the treatment of alopecia areata, a prospective study enrolling 14 patients with 24 patches employed corticosteroid microneedle patches administered once every 4–6 weeks for 24–36 weeks. The results showed that the corticosteroid microneedle patches achieved a mean regrowth score of 4 (maximum score) within 24–36 weeks, with pain scores significantly lower than those of conventional intralesional injection (Ariya-Atthaya et al., 2025). Furthermore, microneedles combined with platelet-rich plasma or concentrated growth factor likewise demonstrated pro-regenerative effects superior to monotherapy (Mishra et al., 2026). With microneedle radiofrequency (MNRF) combined with topical exosome therapy, at a median follow-up of 10 months, results showed significant increases in mean hair density and diameter (Koumprentziotis et al., 2025). For iatrogenic hair loss induced by hyaluronic acid filler injection, a combined regimen of microneedles with CGF and minoxidil also achieved satisfactory hair regeneration outcomes (Xie et al., 2024; Jia et al., 2025). However, most of these studies are small, treatment parameters are inconsistent, and the overall quality of evidence is low (English et al., 2022).

In summary, microneedle technology has emerged as a delivery platform with considerable translational promise in the field of hair loss treatment. Current research centers on material optimization and configuration design of microneedles, intelligent drug release, and integrative applications with nanotechnology, biologics, and cell therapy. Concurrently, clinical studies on microneedle-mediated drug delivery are progressively advancing, preliminarily confirming their efficacy and safety in hair loss treatment. However, the widespread clinical implementation of microneedles still faces several challenges. First, cutting-edge directions in current preclinical research, such as microneedle delivery of nanomedicines, engineered extracellular vesicles, and stem cell derivatives, still confront clinical translational barriers related to scalable manufacturing, stable storage, and appropriate regulatory oversight (Gong et al., 2026). Second, clinical trials conducted to date remain predominantly focused on microneedle delivery of, or combination with, conventional chemical drugs, and generally suffer from limitations such as small sample sizes and short follow-up durations, requiring further improvement in the level of evidence. Additionally, the standardization of administration parameters (e.g., needle length, density, treatment intervals) and the evaluation of long-term safety for different microneedle types warrant further investigation. In the future, by strengthening clinically translation-oriented basic research and advancing large-sample multicenter randomized controlled trials, microneedle therapy is expected to become a routine option in the clinical management of hair loss.

## 4 Regenerative therapies

Regenerative therapies activate epithelial-mesenchymal interactions and improve the hair follicle microenvironment through cell transplantation or the administration of cell-derived secretory products, thereby initiating hair follicle regeneration. This chapter will introduce the roles of platelet derivatives, cell transplantation, and stem cell-derived exosomes in hair regeneration therapy.

### 4.1 Platelet derivatives

Platelet derivatives are a class of preparations obtained from autologous blood that are enriched in platelets and various bioactive molecules. Based on differences in preparation methods and product forms, the main types currently applied in hair loss treatment include: platelet-rich plasma (PRP), platelet-rich fibrin (PRF), concentrated growth factor (CGF), as well as their cell-free derivatives such as platelet lysate (PL) and platelet-derived extracellular vesicles (PEVs). Among these, PRP is high-concentration platelet plasma obtained by centrifuging autologous whole blood, with a platelet concentration typically 3–5 times that of whole blood (Zhong et al., 2026). Both PRF and CGF are fibrin network concentrates obtained through specific centrifugation protocols without the addition of anticoagulants; CGF contains higher concentrations of growth factors and CD34^+^ stem cells (Sharma et al., 2024; Zhou et al., 2025). PL is a growth factor mixture obtained by lysing platelets through methods such as freeze-thawing, whereas PEVs are nanoscale vesicles released from platelets (Lu C. et al., 2025; Wang L. et al., 2025).

The primary mechanism by which platelet derivatives promote hair follicle regeneration lies in the multiple growth factors they release. Platelet α-granules store platelet-derived growth factor (PDGF), vascular endothelial growth factor (VEGF), transforming growth factor-β (TGF-β), epidermal growth factor (EGF), fibroblast growth factor (FGF), insulin-like growth factor-1 (IGF-1), and others. These factors can activate the Wnt/β-catenin pathway, the MAPK/ERK pathway, and the Akt pathway through paracrine mechanisms, promoting the transition of hair follicles from telogen to anagen (Lu C. et al., 2025; Mao et al., 2026) and stimulating dermal papilla cell proliferation (Li et al., 2012). In addition, platelet derivatives can promote perifollicular angiogenesis (Zhong et al., 2026), enhance autophagy levels in hair follicle cells to delay follicle aging (Xiao et al., 2026), and improve the perifollicular immune microenvironment by modulating the levels of inflammatory cytokines such as IL-6 and TNF-α (Vrapcea et al., 2025). Due to differences in preparation methods, variations exist in the active component composition of different platelet derivatives, which may lead to differing clinical efficacy; however, systematic comparative studies are currently lacking.

Platelet derivatives have demonstrated therapeutic potential across multiple types of hair loss (Jafarzadeh et al., 2024). PRP is currently the most extensively studied platelet derivative. Multiple randomized controlled trials (RCTs) and meta-analyses have confirmed that PRP can significantly increase hair density and diameter in patients with androgenetic alopecia, with high patient satisfaction (Anitu et al., 2025; Yuan et al., 2024). Nevertheless, these studies differ considerably in preparation methods, injection frequency, and follow-up duration, and most do not indicate whether the reported changes in hair density or diameter represent clinically visible improvements (Gentile and Garcovich, 2020). In the treatment of alopecia areata, PRP has similarly demonstrated efficacy superior or non-inferior to intralesional corticosteroid injection (Aydogan et al., 2025). Some studies suggest that PRF and CGF may have superior therapeutic effects. A multicenter randomized trial comparing the efficacy of PRP, injectable platelet-rich fibrin (i-PRF), and CGF in patients with female pattern hair loss showed that both the i-PRF group and the CGF group outperformed the PRP group in increasing the number of terminal hairs, with the i-PRF group having the lowest incidence of adverse events (Li et al., 2026). Another retrospective study comparing the efficacy of CGF and PRP in male and female pattern hair loss demonstrated that CGF treatment yielded superior results in total hair count, hair shaft diameter, and global aesthetic improvement scores (Garg and Thirumalaiswamy, 2026).

Studies have shown that combining platelet derivatives with other therapies can achieve superior efficacy. For example, PRP combined with topical minoxidil or basic fibroblast growth factor yields better efficacy than minoxidil alone (Yao et al., 2024; Xia et al., 2025). In the perioperative period of hair transplantation, platelet derivatives have also demonstrated clear adjunctive value. A prospective study demonstrated that the use of PRP after follicular unit excision (FUE) increased hair density by 25% at 6 months post-surgery compared with the surgery-only group (Saxena et al., 2026). Another randomized trial also confirmed that CGF-assisted FUE can significantly increase the proportion of terminal hairs and hair density (Wang Q. et al., 2026). Furthermore, PRP can accelerate wound healing at the FUE donor site (Zontos, 2026).

Although platelet derivatives have demonstrated encouraging clinical prospects, their application lacks standardized preparation and administration protocols. Parameters such as the number of centrifugation steps, centrifugal force and duration, and whether activators are used during preparation (Ghanem et al., 2025), as well as injection dose, frequency, interval, and delivery method during treatment, all lack unified standards (Lu P. et al., 2025). These variations make it difficult to directly compare results across different studies, thereby delaying the establishment of clinical consensus and optimal protocols. In addition, individual factors such as patient age, severity of hair loss, and systemic inflammatory status may also influence treatment response (Vrapcea et al., 2025; Brinks et al., 2025), suggesting that patient selection and individualized regimen development may be warranted in the future.

To address the above limitations, future research should strive to achieve breakthroughs in the following areas. For the establishment of standardized preparation and administration guidelines, further large-sample, multicenter randomized controlled trials should be conducted. For the pain, discomfort, and infection risk associated with conventional injection-based administration, engineered delivery systems may offer superior solutions. For example, machine learning-driven formulation optimization technology has been used to prepare PRP-loaded microneedles with both high mechanical strength and rapid dissolution properties; these microneedles can sustain the release of growth factors for 30 days and demonstrate superior efficacy to daily minoxidil administration in an AGA mouse model (Yan et al., 2025). Another study developed a PRP lysate-loaded hyaluronic acid microneedle patch that can effectively promote hair follicle entry into the anagen phase while enabling painless transdermal delivery, with a dosing frequency lower than that of minoxidil (Suo et al., 2025). These attempts provide feasible solutions for improving patient compliance and enhancing drug delivery efficiency.

### 4.2 Cell transplantation (cell therapy)

Cell transplantation aims to reconstruct the epithelial-mesenchymal interactions (EMI) required for hair follicle regeneration by transplanting cells with hair-inductive capacity into areas of hair loss, thereby initiating the formation of new hair follicles at the source (Sung, 2023).

A diverse range of cell types can be used for transplantation, including dermal papilla cells, dermal sheath cup cells, hair follicle stem cells, and adipose-derived stem cells, all with the goal of replenishing the mesenchymal or epithelial components of the hair follicle. For instance, dermal papilla cells (DPCs) co-transplanted with epidermal cells can induce *de novo* hair follicle formation (Kang et al., 2012). Dermal sheath cup cells (DSCCs) are homologous to DPCs and retain their viability after expansion. A Phase II clinical trial demonstrated that low-dose injection of autologous DSCCs significantly improved hair density and diameter in patients with androgenetic alopecia at 6–9 months (Tsuboi et al., 2020); this therapy was approved in Japan in 2024, becoming the world’s first regulatory-approved hair follicle cell therapy (Kim et al., 2025).

Hair follicle stem cells (HFSCs) or epidermal stem cells (ESCs) can serve as the epithelial component and be co-transplanted with mesenchymal cells. Pre-assembling HFSCs and DPCs at a defined ratio into hair follicle germs (HFGs) prior to transplantation can significantly improve the efficiency of hair follicle formation (Toyoshima et al., 2012). Furthermore, mixing human foreskin-derived ESCs with DPCs and injecting them into the dorsal skin of nude mice resulted in the formation of structurally complete hair follicles within 14 days (Zhang et al., 2020). Adipose-derived stem cells (ASCs) and the stromal vascular fraction (SVF) primarily exert their effects through paracrine mechanisms. ASCs can secrete VEGF, HGF, and other factors to promote angiogenesis and improve local blood supply (Lu et al., 2020). SVF, as a mixed cell population containing ASCs, endothelial cells, and other cell types, can create a pro-regenerative microenvironment for hair growth through synergistic effects. A clinical study showed that 6 months after scalp injection of autologous SVF in patients with alopecia areata, the proportion of anagen hair follicles increased from approximately 60%–93% (Anderi et al., 2018). In patients with androgenetic alopecia, SVF injection also significantly increased hair density and diameter (Perez-Meza et al., 2017).

### 4.3 Exosomes

Although cell therapy has demonstrated therapeutic potential, its clinical application still faces challenges such as low cell survival rates, tumorigenic risk, and difficulties in standardization (Xu et al., 2025). Exosomes are nanoscale vesicles with a diameter of 30–150 nm secreted by cells, capable of carrying a variety of bioactive molecules including proteins, lipids, mRNAs, and microRNAs [Exosomes]. Compared with living cells, exosomes offer advantages such as lower immunogenicity, easier storage and standardization, and no tumorigenic risk, making them ideal carriers for cell-free therapy (Kost et al., 2022).

Exosomes derived from multiple stem cell sources have demonstrated hair follicle regeneration-promoting effects. Studies have shown that exosomes derived from dermal papilla cells, dermal sheath cup cells (DSCCs), human umbilical cord mesenchymal stem cells (hUCMSCs), and human amniotic mesenchymal stem cells can all activate the Wnt/β-catenin pathway (Kost et al., 2022; Liu et al., 2024; Hu et al., 2020; Wang XY. et al., 2025). Pretreatment of hUCMSCs with the Wnt pathway agonist lithium chloride can enrich specific molecules such as miR-146a-5p in exosomes, thereby enhancing the activation efficiency of the Wnt signaling pathway (Wang Z. et al., 2025). Adipose-derived mesenchymal stem cell-derived exosomes have been demonstrated to upregulate PDGF and VEGF expression while downregulating TGF-β1 levels, thereby promoting hair follicle regeneration *in vivo* (Wu et al., 2021; Ma et al., 2025).

The efficacy of exosomes has also been confirmed by clinical studies. An observational study investigated the efficacy of rose stem cell-derived exosomes in the treatment of gray hair; the results showed hair repigmentation after treatment, with effects observed after an average of 2.4 treatment sessions and maintained for 4.7 months (Lueangarun et al., 2025). In a clinical trial involving androgenetic alopecia patients, those receiving adipose-derived mesenchymal stem cell-derived exosome treatment demonstrated significantly increased total hair density at 24 weeks, higher patient satisfaction, and no serious adverse reactions (Lee et al., 2024). Current exosome evidence remains preliminary, with few randomized controlled trials and a predominance of single-arm, retrospective, or case-based studies that have small samples and short follow-up periods (Al Ameer et al., 2025; Gupta et al., 2023).

To enhance the targeting and therapeutic efficacy of exosomes, researchers have developed various engineering strategies. For example, combining dermal papilla cell-derived exosomes with the collagenous sequence of human type XVII collagen can significantly enhance the migration and proliferation capacity of hair follicle stem cells by regulating the hsa-novel-238a-CASP9 axis (Zhao et al., 2025b). Another study developed a minoxidil-loaded engineered exosome biopotentiated hydrogel that combines the skin penetration capacity of flexible liposomes with the targeting ability of exosomes; in an androgenetic alopecia mouse model, this hydrogel demonstrated a faster hair regrowth rate compared with free minoxidil without causing significant skin irritation (Zhang H. et al., 2024).

## 5 Hair follicle biofabrication

For patients with cicatricial alopecia in which hair follicles have been completely destroyed, or those with severe depletion of donor hair, neither pharmacological treatment nor autologous hair transplantation is feasible, and an additional source of hair follicle grafts is required. In recent years, significant progress has been made in constructing hair follicle units with intact structure and function using epithelial and mesenchymal cells *in vitro*. These in vitro-constructed hair follicles not only provide potential hair follicle graft donors for regenerative medicine but also serve as important platforms for studying hair follicle developmental mechanisms, establishing disease models, and screening drugs. This chapter will outline the current major *in vitro* hair follicle construction systems, with an emphasis on their application status in mechanistic research, disease modeling, drug screening, and regenerative medicine, and will discuss the key challenges facing clinical translation (Figure 2).

**FIGURE 2:**
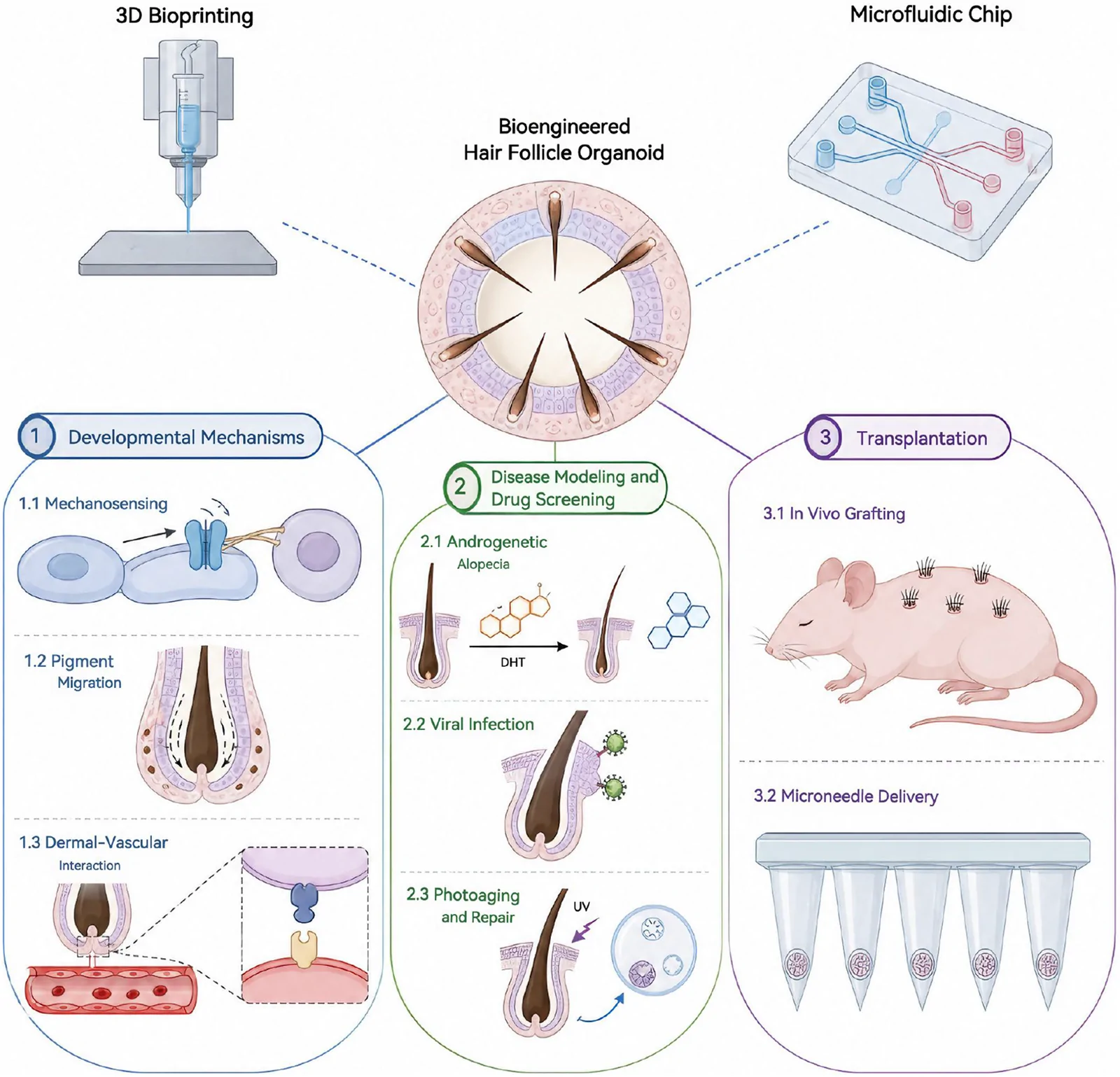
Applications of bioengineered hair follicles in mechanism, modeling, and transplantation.

### 5.1 Overview of biofabricated hair follicle types

Current strategies for constructing hair follicles *in vitro* can be broadly categorized, depending on their reliance on cell self-organization capacity, into hair follicle organoids and engineered constructs based on biofabrication technologies.

Hair follicle organoids are miniature hair follicle structures spontaneously formed by exploiting the self-organizing capacity of epithelial and mesenchymal cells under three-dimensional culture conditions. The underlying principle is based on the fundamental laws of hair follicle morphogenesis during embryonic development, whereby epithelial and mesenchymal cells, driven by their mutual interactions and specific microenvironmental signaling cues, can autonomously arrange themselves into ordered hair follicle structures. The epithelial component for constructing hair follicle organoids can be derived from hair follicle outer root sheath keratinocytes, hair follicle stem cells, or epidermal stem cells, while the mesenchymal component most commonly employs dermal papilla cells or dermal fibroblasts. In recent years, human induced pluripotent stem cells (hiPSCs) have become an important cell source for organoids owing to their unlimited proliferative capacity and multilineage differentiation potential (Zhang YX. et al., 2024; Mostina et al., 2025). Regarding culture systems, researchers typically mix dissociated epithelial and mesenchymal cells at a defined ratio, embed them in extracellular matrices such as Matrigel or collagen, and perform three-dimensional suspension culture in specific culture media (Kageyama et al., 2022a). The Koehler team established a representative hiPSC-derived culture protocol for hair-bearing skin organoids. By temporally regulating TGFβ, BMP, and FGF signaling pathways to first induce the differentiation of surface ectoderm and cranial neural crest cells, followed by approximately 4–5 months of continuous culture, they ultimately obtained cystic skin organoids comprising stratified epidermis, pigmented hair follicles, sebaceous glands, and Merkel cells, with a cellular composition highly similar to that of human fetal facial skin at 18 weeks of gestation (Lee et al., 2022; Lee et al., 2020). However, these organoids form an inverted cystic structure with the epidermis on the inside and the dermis on the outside during culture, with hair shafts growing inward toward the cystic lumen, which is unfavorable for the natural excretion of keratinous debris and also limits their application in barrier function studies and drug testing. To improve the efficiency and quality of hair follicle induction, subsequent studies have explored various culture optimization strategies, such as adding low-concentration Matrigel or Y27632 to modulate cell sorting behavior, employing air-liquid interface culture to promote planar epidermal development and outward hair follicle growth, and using microwell array plates to achieve standardized preparation of single hair follicle-containing organoids (Kageyama et al., 2022a; Kageyama et al., 2022b; De Henau et al., 2025; Sun J. et al., 2025; Jung et al., 2022). The above studies have laid the foundation for the application of hair follicle organoids in disease modeling and drug screening.

Engineered hair follicle constructs based on biofabrication technologies primarily employ engineering techniques such as 3D bioprinting and microfluidics to predefine the spatial arrangement of cells, thereby guiding the formation of hair follicle units. 3D bioprinting technology uses pre-designed computer models to deposit bioinks containing dermal papilla cells, epidermal cells, and fibroblasts layer by layer, constructing skin-hair follicle composite tissues with biomimetic spatial architecture. For example, using gelatin-alginate hydrogel as the bioink and adopting a layered printing strategy—with fibroblasts simulating the dermal matrix in the bottom layer, dermal papilla cells printed in a dot-matrix pattern in the middle layer, and epidermal cells covering the top layer—hair follicles with directional growth characteristics can be regenerated *in vivo* (Motter et al., 2023; Kang et al., 2023). Microfluidic technology enables precise manipulation of fluid behavior within micron-scale channels. By simultaneously introducing collagen solutions separately encapsulating epithelial cells and mesenchymal cells into a flow-focusing microfluidic device, double-faced microbeads each containing one cell type on either side can be formed. After 3 days of culture, the microbeads contract into compact hair follicle germs due to cellular traction forces, with a production rate reaching 300 germs per minute (Sugiyama et al., 2024; Kageyama et al., 2018). Compared with organoids, hair follicle construction strategies based on biofabrication technologies guide tissue formation through predefined spatial constraints, offering advantages such as higher standardization and suitability for scalable production. However, they impose higher demands on equipment and materials, and currently, the structural integrity and functional maturity of regenerated hair follicles still fall short of those achieved by self-assembled organoids. In addition, organ-on-a-chip technology maintains the hair follicle microenvironment on miniature chips through microfluidic perfusion and biomimetic materials, supporting the long-term dynamic culture of *ex vivo* hair follicle units and providing a new *in vitro* platform for drug screening (Jeong et al., 2023; Ataç et al., 2013).

These approaches differ in structural complexity, throughput, standardization, and translational readiness, as summarized in Table 2.

**TABLE 2:** Comparative evaluation of *in vitro* hair follicle construction technologies.

Technology	Cell source	Structural complexity and integration	Throughput	Standardization, scalability, and cost	Maturity	Best use	Translational bottleneck	References
Hair follicle organoids	Epithelial cells (ORS keratinocytes, HFSCs or epidermal stem cells) and mesenchymal cells (DPCs or fibroblasts) HiPSC/iPSC-derived cells	Can form stratified epidermis, pigmented follicles, sebaceous glands, Merkel cells and sensory neurons in long-term culture Vascularized and immune-containing organoids are emerging. Some protocols form inverted cystic structures	Low to moderate Culture often requires weeks to months Batch variation is substantial	Difficult Undefined matrices, long culture and high cost limit reproducibility and scale-up	High biological fidelity Low scalability	Developmental mechanisms, disease modeling, drug screening, pigmentation and immune-mediated alopecia	Long culture time, low survival after transplantation, limited reproducibility and scale-up	(Sung, 2023; Kang et al., 2012; Tsuboi et al., 2020; Kim et al., 2025; Kost et al., 2022; Liu et al., 2024; Hu et al., 2020; Wang et al., 2025b; Wang et al., 2025c; Wu et al., 2021; Ma et al., 2025; Lueangarun et al., 2025; Lee et al., 2024)
3D bioprinting	DPCs, epidermal cells and fibroblasts Endothelial cells and melanocytes can be incorporated	Predefined spatial architecture Layered skin-hair constructs and directional follicle growth Vascular networks are emerging	Moderate Depends on printer configuration and automation	Higher reproducibility than organoids, but equipment, bioink and material costs are high Bioink standardization is needed	Preclinical	Spatially controlled follicle construction, skin-hair composites and transplantable grafts	Limited follicle maturity, vascularization, automation and cost	(Toyoshima et al., 2012; Zhang et al., 2020; Al Ameer et al., 2025; Gupta et al., 2023)
Microfluidics	Epithelial and mesenchymal cells Used to generate hair follicle germs	Double-faced microbeads and compact hair follicle germs Tissue complexity remains limited	High One system produced approximately 300 hair follicle germs per minute	Good throughput and reproducibility Device fabrication and biomaterial costs remain barriers	Preclinical	Large-scale hair follicle germ production, screening and combinatorial culture	Limited structural and functional maturity Downstream transplantation is not established	(Lu et al., 2020; Anderi et al., 2018; Liu et al., 2024; Gupta et al., 2023)
Organ-on-a-chip	Skin or hair follicle cells, tissue explants and iPSC-derived cells	Dynamic perfusion maintains the follicular microenvironment Immune and vascular components can be integrated, but full organ complexity is limited	Moderate Optimized for mechanistic studies and drug screening rather than mass production	Suitable for standardized assays Not designed for scalable graft production	Preclinical research tool	Dynamic culture, drug screening, mechanism studies and toxicity testing	Not a transplant source Limited scale and complex validation	(Perez-Meza et al., 2017; Xu et al., 2025; Gupta et al., 2023; Zhao et al., 2025b)

### 5.2 Biofabricated hair follicles for studying hair development and regeneration mechanisms

The self-organizing properties of organoids enable them to recapitulate key steps in hair follicle development, making them suitable as models for studying hair follicle developmental mechanisms. For instance, during the self-organization of skin organoids, researchers discovered that the contractile forces of dermal cells drive them to envelop epidermal aggregates, thereby initiating the initial epithelial-mesenchymal interaction. Epidermal cells sense tensile forces through the mechanosensor Piezo1, which further regulates dermal-epidermal adhesion, forming a mechano-chemical coupling circuit that governs hair follicle development (Lei et al., 2023; Wang et al., 2023).

In studies on the mechanisms of hair pigmentation, researchers constructed skin organoids using dissociated epidermal cells, dermal cells, and melanocyte progenitor cells, successfully reconstructing pigmented hair *in vitro* and revealing the regulatory mechanisms of the pigmentation process: dermal fibroblasts maintain melanocyte survival through the COL6A3-CD44 pathway, after which the hair follicle bulge guides the directional migration of melanocytes to their stem cell niche via SEMA3C-NRP1 signaling, thereby enhancing hair shaft pigmentation (Li et al., 2025). An *in vitro* hair pigmentation model constructed based on hair follicle organoids also enables functional validation of genes related to melanosome production and transport, providing a platform for research on the mechanisms of gray hair and screening of interventional drugs (Tu et al., 2025).

The interaction between hair follicles and blood vessels is of great significance for hair regeneration. Studies have shown that bidirectional signaling crosstalk exists between dermal papilla cells and endothelial cells (Zhou et al., 2024). EDN1 secreted by endothelial cells activates taurine metabolism in dermal papilla cells to promote hair growth, while CTF1 released by the dermal papilla activates α-linolenic acid metabolism in endothelial cells to promote angiogenesis. This interaction weakens during aging and may represent one of the important mechanisms underlying senile alopecia (Zhou et al., 2024).

### 5.3 Biofabricated hair follicles for disease modeling and drug screening

*In vitro* hair follicle constructs provide an experimental platform that more closely approximates human physiological conditions for mechanistic studies of hair loss-related diseases and high-throughput evaluation of therapeutic drugs (Ataç et al., 2020; Xie et al., 2025). In studies on androgenetic alopecia, researchers treated embryonic stem cell-derived hair follicle organoids with dihydrotestosterone and successfully established an androgenetic alopecia model, characterized by a reduced number of pigmented hair shafts and downregulated expression of anagen markers. Co-treatment with minoxidil and soybean germ extract reversed the above pathological changes, validating the feasibility of this organoid model for AGA drug screening (Kyun et al., 2026). Using hair follicle organoids for *in vitro* screening, researchers have also identified multiple small-molecule compounds with hair growth-promoting activity. For example, cinnamic acid activates the hair-inductive function of dermal papilla cells by upregulating oxytocin receptor expression, promoting hair follicle germ elongation (Kageyama et al., 2024; Kageyama et al., 2023). Serotonin promotes dermal papilla cell proliferation and hair shaft elongation through its receptors (Kageyama et al., 2025). These findings provide new candidate targets for hair loss treatment.

Skin organoids have also been employed to construct pathological models of infection-related hair loss (Aliyazdi et al., 2024). One study established a SARS-CoV-2 infection model using hiPSC-derived skin organoids and found that SARS-CoV-2 can directly infect KRT17-positive hair follicle stem cells, leading to hair follicle structural destruction and inhibition of hair growth, providing direct evidence to explain the acute telogen effluvium observed in COVID-19 patients (Ma et al., 2022).

Organoid models can likewise be used to study hair follicle damage induced by ultraviolet radiation. After exposure to simulated solar ultraviolet radiation, human skin organoids containing hair follicles exhibited features such as hair follicle structural destruction and premature catagen entry. Treatment with human umbilical cord blood mesenchymal stem cell-derived exosomes alleviated the above damage and promoted hair follicle regeneration (Kim et al., 2024).

### 5.4 Biofabricated hair follicles as donor sources for hair transplantation

In vitro-constructed hair follicles can serve as a renewable donor graft source for patients with hair follicle deficiency, an application that has been fully validated in animal experiments. Hair follicle germs or skin organoids constructed *in vitro*, when transplanted onto the dorsal skin of immunodeficient mice, can form regenerated hair follicles with intact structure that are capable of connecting to host arrector pili muscles and nerve fibers and repeating normal hair cycles (Toyoshima et al., 2012). One study used cells derived from androgenetic alopecia patients to regenerate pigmented human hair follicles in nude mice, achieving a density of approximately 124 hairs/cm^2^ (Toyoshima et al., 2012).

How to efficiently deliver in vitro-constructed hair follicle units *in vivo* and ensure their survival is a problem that needs to be addressed for their clinical translation. Conventional surgical transplantation is not only procedurally complex and associated with significant trauma, but post-transplantation support materials such as hydrogels may also restrict organoid growth and the emergence of hair shafts through the skin. To address this issue, researchers developed a matrix-free organoid delivery system based on cryomicroneedle array patches. This approach circumvents the inhibitory effect of hydrogels on organoids, achieving a success rate of hair-penetrating skin regeneration exceeding 86% within 15 days, providing a novel solution for minimally invasive and highly efficient hair transplantation (Wang S. et al., 2026).

Immune rejection represents another obstacle facing allogeneic transplantation. In this regard, the use of patient-specific autologous iPSCs to differentiate hair follicle organoids can fundamentally avoid immune rejection and may, in theory, enable personalized hair follicle regenerative therapy (Zhang YX. et al., 2024; Vatanashevanopakorn and Sartyoungkul, 2023).

## 6 Discussion

The research focus of hair follicle regenerative medicine has shifted toward the active modulation and functional reconstruction of the hair follicle regenerative microenvironment, with the ultimate goal of achieving sustained, cyclical hair follicle regeneration. Although considerable progress has been made in recent years in areas such as hair follicle-targeted drug delivery, biomaterials, regenerative medicine, and tissue engineering, the path to clinical translation still faces numerous challenges.

Table 3 provides a comparative overview of the therapeutic strategies discussed in this review. Table 4 summarizes the clinical studies supporting this comparison. Overall, these strategies differ considerably in clinical evidence, regenerative potential, and translational status. Microneedles and platelet derivatives have accumulated comparatively more clinical data, although existing studies mainly show improvement in follicles that remain viable and do not yet establish that new follicles can be generated. Exosomes have been studied less extensively, and preliminary results suggest a favorable safety profile, whereas their efficacy and protocol consistency remain uncertain. Most studies use hair density or diameter as endpoints, but rarely indicate whether these changes translate into clinically visible improvements (Kaiser et al., 2023; Behrangi et al., 2026). In summary, microneedles and platelet derivatives are already in clinical use, although treatment courses are long and patient satisfaction remains limited. Hair follicle organoids developed from stem cell research are still at the laboratory stage, but are regarded as a highly promising therapeutic strategy.

**TABLE 3:** Comparative evaluation of therapeutic strategies.

Strategy	Core function and mechanism	Evidence and translational status	Efficacy and scope	Main advantages	Main limitations	Standardization/scalability	References
Nanocarriers	Increase solubility, stability and follicular targeting Enable controlled release Some materials have intrinsic antioxidant, anti-inflammatory or enzyme-like activity. Pathways are mainly cargo-dependent	Mostly preclinical No completed human clinical trial with reported results Several registered trials remain unreported	Enhances delivery of drugs, nucleic acids and biologics Mainly maintains or activates existing follicles. Evidence for *de novo* follicle formation is limited	Targeted delivery Reduced systemic exposure Tunable release Can deliver poorly soluble drugs	Batch-to-batch consistency Long-term safety Scale-up Limited clinical evidence Cargo-dependent effects	Challenging Material-dependent Limited consensus on quality attributes	(Correia et al., 2023; Ben-Shaanan et al., 2024; Lee et al., 2026; Fereig et al., 2025; Chang et al., 2025a; Gupta et al., 2023; Koç Babayiğit et al., 2025; Gentile, 2026)
Microneedles	Penetrate the stratum corneum, create microchannels and provide mechanical stimulation Deliver drugs, biologics, cells or exosomes. Biological pathways depend on the delivered cargo and mechanical stimulation	Multiple clinical studies, mostly small Oxford Centre for Evidence-Based Medicine (OCEBM) level 2–3 Early clinical use. Evidence quality is heterogeneous	Increases hair density and diameter Useful as an adjunct to minoxidil, PRP or exosomes Mainly improves surviving follicles. Robust evidence for new follicle formation is lacking	Minimally invasive Repeatable High delivery efficiency Less painful than intralesional injection	Needle length, density and treatment frequency are not standardized Small trials Modest evidence quality	Moderate Device production is feasible, but administration parameters require harmonization	(Ben-Shaanan et al., 2024; Fereig et al., 2026; Zhao et al., 2025a; Thepphankulngarm et al., 2024; Chen et al., 2025a; Ye et al., 2025; Suo et al., 2025; Lu et al., 2025a)
Platelet derivatives	Autologous concentrates supply growth factors and cytokines Modulate angiogenesis, inflammation, autophagy and the perifollicular microenvironment. PRP, PRF and CGF differ in composition	Many RCTs and meta-analyses OCEBM Level 2-4 Widely used clinically but protocols are heterogeneous	Increases hair density and diameter Useful as monotherapy or adjunct to transplantation Effects mainly reflect activation of surviving follicles	Autologous Generally favorable safety Low immunogenicity Readily available in clinical practice	Preparation methods and dosing are inconsistent Variable composition Short follow-up in many studies	Difficult Centrifugation, activation, dose and administration protocols vary No unified standards	(Sun et al., 2026; Xing et al., 2025a; Wan et al., 2026; Ariya-Atthaya et al., 2025; Koumprentziotis et al., 2025; Jia et al., 2025; Zhou et al., 2025; Legiawati et al., 2025)
Cell transplantation	Restores epithelial-mesenchymal interactions and provides paracrine support Includes DPCs, DSCCs, HFSCs, ASCs and SVF.	A few early clinical trials DSCC therapy is approved in Japan OCEBM Level 2-4	Potential to support new follicle formation and improve density or diameter Efficacy varies by cell type and patient	Potential structural regeneration Autologous options Paracrine and immunomodulatory effects	Low cell survival Complex preparation, storage and quality control Tumorigenic and immunogenicity concerns High cost	Poor Cell manufacturing and release testing are complex	(Xiao et al., 2026; Vrapcea et al., 2025; Jafarzadeh et al., 2024; Yuan et al., 2024; Gentile and Garcovich, 2020; Aydogan et al., 2025; Li et al., 2026; Garg and Thirumalaiswamy, 2026)
Exosomes	Cell-free nanoscale vesicles deliver miRNA, proteins and lipids Mediate paracrine signaling, immune modulation and vascular support Can be engineered	Preclinical and early clinical evidence OCEBM Level 2-4 No approved product	Increases hair density and may improve pigmentation Favorable safety signals. Evidence for new follicle formation remains limited	Cell-free Lower immunogenicity than cells Relatively easy storage Engineerable cargo	Source and characterization vary Dosing and potency assays are not standardized Short follow-up Heterogeneous protocols	Manufacturing and quality standards are not established	(Yao et al., 2024; Wang et al., 2026b; Zontos, 2026; Ghanem et al., 2025; Lu et al., 2025b; Brinks et al., 2025; Yan et al., 2025; Abedini et al., 2025; Xue et al., 2025; Brinks et al., 2026)

**TABLE 4:** Summary of clinical evidence from studies published within the past 2 years (2024–2026).

Study	Design	Population	Intervention and comparator	Hair-related outcomes	Follow-up	Evidence level (OCEBM)	Main limitations
(Charoensuksira et al., 2024)	Randomized split-scalp trial	16 patients with androgenetic alopecia (12 men and 4 women)	A light-guiding microneedle patch combined with an LED helmet, applied weekly for 24 weeks. One side of the scalp received green light and the other side red light	Hair density and hair diameter increased significantly on both sides, and the changes did not differ significantly between green and red light	24 weeks	2	Small sample. No untreated control. Both sides received active treatment
(Gohary et al., 2025)	Randomized, single-blind, placebo-controlled trial	80 men with androgenetic alopecia (40 per group)	Topical 1% cetirizine delivered by microneedling compared with saline delivered by microneedling	Hair density improved in 80.0% of patients treated with cetirizine and in 25.0% of patients treated with saline (p = 0.001). Vellus hair was present in 70.0% and 30.0% of patients, respectively (p = 0.001)	8 weeks	2	Short follow-up. Men only
(Lu et al., 2025b)	Randomized split-scalp study	20 patients with androgenetic alopecia	Platelet-rich plasma (PRP) delivered by intradermal injection compared with PRP delivered by microneedling, three sessions 1 month apart	Hair density, hair diameter, single-hair follicle ratio and trichoscopic findings did not differ significantly between the two delivery methods	6 months after the last session	2	Small sample. Split-scalp design. No placebo
(Qiao et al., 2025)	Randomized controlled trial	80 patients with mild-to-moderate patchy alopecia areata (SALT score below 50)	Compound betamethasone given monthly for 3 months by intralesional injection compared with transdermal microneedle delivery	The mean reduction in SALT score was 7.00 ± 4.50 points after injection and 8.08 ± 8.01 points after microneedle delivery, with no significant difference. Remission rates for SALT30, SALT50, SALT75 and SALT90 were 92.5%, 90.0%, 57.5% and 42.5% after injection and 95.0%, 87.5%, 72.5% and 40.0% after microneedle delivery	3 months	2	Only mild-to-moderate patchy alopecia areata was included. Short follow-up
(Chang et al., 2025b)	Three-group comparative clinical study	45 men with androgenetic alopecia (15 per group)	Microneedling plus 5% minoxidil and finasteride (group C) compared with 5% minoxidil alone (group A) and with minoxidil plus finasteride (group B)	Hair density and hair shaft diameter increased in all groups, and the increase was greater in groups B and C than in group A (p < 0.05), with no significant difference between groups B and C. The Norwood–Hamilton grade improved more in group C than in group A (p < 0.01)	1–6 months	3	Small sample. Randomization and blinding not clearly described
(Wan et al., 2026)	Prospective controlled study of three parallel groups	30 patients with androgenetic alopecia or diffuse hair loss (10 per group)	Microneedling followed by topical 2% chitosan compared with 5% topical minoxidil and with an untreated control group	Hair density increased by 30.5% in the chitosan group and by 22.3% in the minoxidil group relative to the untreated control group (p = 0.032 and p = 0.048). The difference between the chitosan and minoxidil groups was not significant (p = 0.62). Hair shaft diameter also improved in both treated groups	6 months	2	Small sample
(Liu et al., 2026)	Prospective, randomized, open-label, single-centre trial	245 women with mild-to-moderate androgenetic alopecia (234 completed the study)	2% topical minoxidil alone compared with minoxidil plus microneedling every 4 weeks and with minoxidil plus microneedling every 2 weeks	The nonvellus hair count in the target area increased significantly in all three groups (p < 0.001), with no significant differences between groups. Hair shaft diameter, vellus hair count and hair density were also comparable between groups	24 weeks	2	Open-label, single-centre design. No patient-reported outcomes
(Nilforoushzadeh et al., 2025)	Phase I randomized clinical trial	40 patients with androgenetic alopecia	PRP injections compared with microneedling, two sessions 1 month apart	Hair count increased by 62.4% after PRP injection (from 17.40 ± 2.07 to 28.26 ± 6.23) and by 88.4% after microneedling (from 14.71 ± 3.99 to 27.71 ± 4.50). Hair thickness increased by 58.6% and 51.3%, respectively. The differences between groups were not significant	2 months after the last session	2	Small sample. Short follow-up
(Abedini et al., 2025)	Randomized, double-blind, split-scalp trial	14 patients with severe alopecia areata	Diphenylcyclopropenone (DPCP) plus PRP on one side of the scalp compared with DPCP plus placebo on the other side, with three PRP injections	7 of 14 patients (50%) achieved more than 50% hair regrowth. SALT and trichoscopy scores decreased in both groups without a significant difference between them	6 months	2	Small sample. PRP did not add benefit to DPCP.
(Xue et al., 2025)	Prospective comparative study	30 patients with androgenetic alopecia (15 per group)	PRP injections combined with drug therapy and follicular unit excision compared with drug therapy and follicular unit excision alone	Follicle survival was 88.42% ± 6.21% with PRP and 78.24% ± 8.31% without PRP at 3 months (p = 0.001), and 82.17% ± 4.14% versus 74.01% ± 5.25% at 6 months (p = 0.002). The time to initial hair growth was 17.67 ± 2.23 days with PRP and 20.07 ± 2.82 days without PRP (p = 0.015). The hair pull test was negative in 13 of 15 and in 8 of 15 patients, respectively (p = 0.046)	6 months	2	Small sample. Short follow-up. Units and long-term durability incompletely reported
(Brinks et al., 2026)	Retrospective study	55 patients with androgenetic alopecia (6 received PRP alone and 49 received PRP plus systemic therapy)	PRP alone compared with PRP combined with low-dose oral minoxidil, spironolactone, finasteride or dutasteride	Hair density increased by 6.06 hairs per cm^2^ more and hair width by 3.59 µm more in the combination group than in the monotherapy group, but neither difference was statistically significant (p = 0.703 and p = 0.460)	Variable	3	Very small monotherapy group. Retrospective design
(Li et al., 2026)	Prospective, multicentre, randomized trial	74 women with female pattern hair loss	Four sessions of PRP compared with injectable platelet-rich fibrin (i-PRF) and with concentrated growth factor (CGF), given at 4-week intervals	Nonvellus hair count in the target area increased by 14.53% ± 8.65% with PRP, by 26.54% ± 11.46% with i-PRF and by 27.11% ± 14.80% with CGF, and the increase was significantly greater with i-PRF and CGF than with PRP (p < 0.05). Hair diameter improved in all groups without significant differences between them	24 weeks	2	No placebo group
(Mishra et al., 2026)	Prospective, randomized, double-blind, split-scalp trial	20 patients with androgenetic alopecia (12 men and 8 women)	PRP combined with microneedling compared with PRP plus CGF combined with microneedling	On the side treated with PRP plus CGF, hair count increased from 88.15 ± 10.35 to 135.95 ± 14.46 per 0.65 cm^2^ and hair density from 136.2 ± 8.27 to 162.5 ± 8.1 hairs per cm^2^, which was greater than with PRP alone (p < 0.05)	6 months	2	Small sample. Split-scalp design
(Janaani et al., 2025)	Randomized, observer-blinded trial	75 patients with androgenetic alopecia (60 completed the study)	Low-dose oral minoxidil compared with topical minoxidil and with PRP plus topical minoxidil	Improvement by one grade in alopecia severity occurred in 25%, 10% and 25% of patients, respectively (p = 0.62). The median increase in terminal hair density was 47.5%, 48% and 57%, favouring PRP plus topical minoxidil over the other two groups (p = 0.03 and p = 0.02). Vellus hair density decreased by 26%, 21% and 19%	32 weeks	2	15 of 75 patients dropped out. Differences between groups were modest
(Koç Babayiğit et al., 2025)	Retrospective comparative study	45 patients with androgenetic alopecia (53.3% women)	5% minoxidil compared with PRP and with their combination	Hair count, hair density, terminal hair count and anagen ratio improved significantly in the PRP and combination groups (p < 0.05). Vellus hair count and density decreased significantly in the combination group (p < 0.05)	6 months	3	Retrospective design. Non-randomized groups
(Aydogan et al., 2025)	Retrospective comparative study	75 patients with alopecia areata (36 received PRP and 39 received intralesional steroid)	Three sessions of intralesional PRP compared with intralesional steroid injections	SALT scores at 3 and 6 months were significantly lower in the PRP group than in the steroid group (p = 0.038 and p < 0.001). Among patients who received PRP, the response was very good in 83.4%, good in 8.4%, partial in 2.7% and absent in 5.5%, and relapse occurred in 5.9%	6 months	3	Retrospective design. Small sample
(Gentile, 2026)	Prospective randomized evaluator-blinded trial with a delayed-treatment control group, followed by a long-term observational extension	234 patients with androgenetic alopecia (135 men and 99 women)	Three sessions of autologous non-activated PRP given intradermally at 30-day intervals compared with a delayed-treatment control group that started the same protocol after the initial observation phase	At 12 months, hair density had increased by 31.4%, hair thickness by 26.8% and total hair count by 29.7% (p < 0.001). Hair density remained above baseline at 36 months (24.1%) and at 60 months (17.3%), and was 11.2% above baseline at 120 months in patients who received maintenance treatment	Up to 120 months	2	Randomized comparison limited to the initial phase. Long-term data observational. 39.3% of patients lost to follow-up by 10 years
(Legiawati et al., 2025)	Comparative three-group clinical study	60 patients with androgenetic alopecia (20 per group)	Minoxidil compared with adipose-derived stem cell secretome and with their combination, given at weeks 0, 4 and 8	Hair growth parameters improved significantly in all groups (p < 0.05), with the greatest improvement in the combination group	12 weeks	3	Short follow-up. No placebo group

When the clinical translation of these strategies is considered, manufacturing and regulatory issues become more prominent. Production under good manufacturing practice (GMP) requires defined critical quality attributes and batch-to-batch consistency within closed systems. These requirements are particularly challenging for cell, exosome, and organoid products intended for hair follicle regeneration. Cell products must retain hair-inductive capacity during expansion, and their potency cannot be assessed by viability or surface markers alone. Assays for follicle induction, hair shaft formation, and cycle initiation are also needed. Exosome products require standardized source cells and characterization of hair growth-promoting cargo, together with evidence that they reach the perifollicular niche after delivery. For organoids, hair follicle density, structural maturity, pigmentation, and the ability to sustain cyclical growth vary between batches, and these properties remain difficult to quantify with current release assays. Product standardization therefore depends on hair follicle-specific potency assays, stability data, and release criteria. Regulatory pathways remain inconsistent because hair follicle products may be classified as cell therapies, biologics, or device-drug combinations, and the approved hair follicle cell therapy in Japan remains a relatively isolated example (Kim et al., 2025). Although large-scale manufacturing of biological products for hair follicle regeneration is still at an early stage, dermatology provides more established examples that can inform this process. GMP-manufactured ABCB5+ dermal mesenchymal stromal cells have been produced through validated processes (Kerstan et al., 2021; Kerstan et al., 2022). Autologous skin substitutes in Europe are also regulated as advanced therapy medicinal products (ATMPs), with release testing performed at each critical production stage, including biopsy reception, cell isolation, expansion, passaging, and final product preparation (de la Cruz Valencia et al., 2025; Auxenfans et al., 2025). These examples provide a practical reference for the manufacturing and regulatory requirements that hair follicle regenerative products may also face. For hair follicle products, manufacturing costs and cold-chain logistics remain additional barriers to translation (Cao et al., 2025).

Long-term safety data remain limited. Key concerns include the tumorigenic potential of cell products, the immunogenicity of allogeneic products, the accumulation of nanomaterials, and the cumulative toxicity of repeated administration (Al Ameer et al., 2025). These concerns are particularly relevant for products intended for repeated or long-term use in a chronic condition such as hair loss.

Despite some progress, several fundamental questions remain. Current clinical evidence comes mainly from androgenetic alopecia, whereas data for other types, such as alopecia areata and scarring alopecia, remain limited, and there is little basis for selecting a strategy according to the type of hair loss or the condition of the remaining follicles. It remains unclear whether these strategies can induce entirely new hair follicles in scalp in which follicles have been destroyed and replaced by fibrous tissue, as only sporadic findings from animal studies are available. Whether the effects can be maintained across multiple hair cycles and whether relapse occurs after treatment is discontinued cannot be answered because follow-up in existing studies is generally short, and pharmacological treatments often require long treatment courses with limited efficacy. Outcome measures also differ across studies, and most report changes in hair density or diameter without established criteria for whether such changes are clinically perceptible to patients. At the mechanistic level, the process by which the microenvironment initiates hair follicle cycle transition is still incompletely understood, and the differences between human hair follicles and mouse models, the signaling exchange between grafts and the host microenvironment after transplantation, and the cellular origin of newly formed hair follicles remain unclear. Hair follicle organoids constructed *in vitro* require prolonged culture and show low survival rates, and whether they can sustain cyclical growth over the long term has yet to be demonstrated.

To address these questions, future research could proceed along three lines. For completely atrophic or fibrotic scalp, tissue engineering strategies are needed to reconstruct the follicular niche and support *de novo* follicle formation. For treatment durability, long-term follow-up is needed to determine whether effects can persist across multiple hair cycles and whether repeated intervention is required. For patient selection, single-cell and spatial approaches may help identify predictive biomarkers, and standardized criteria for clinically meaningful outcomes need to be established. These directions also suggest that individualized treatment and engineered follicle construction may deserve further development. In addition, several emerging pharmacotherapies deserve attention, including androgen receptor degraders, prolactin receptor antibodies, metabolic activators, and agents that modulate longevity pathways (Agrawal et al., 2026). Gene editing is being used to test the functions of genes involved in follicle development and cycling and may offer long-term options for hereditary hair loss (Bellani et al., 2025). Single-cell and spatial transcriptomic approaches can map the cellular and signaling changes in the follicular microenvironment and help identify new therapeutic targets (Zhao et al., 2023; Lan X. et al., 2026). Photobiomodulation may also serve as an adjunct to combination therapy. Existing studies indicate that it can activate β-catenin in hair follicle stem cells through reactive oxygen species and paracrine Wnts (Jin et al., 2021), while red light promotes dermal-epidermal remodeling through TGFβ/AKT-mediated collagen dynamics (Chang H. et al., 2025). These approaches may provide new targets, evaluation tools, or combination options for current strategies.

## 7 Conclusion

The goal of functional hair follicle regeneration has gradually shifted from delaying follicle miniaturization toward reconstructing follicles that can undergo sustained cyclical growth. Future research will need to accumulate more evidence in regenerative medicine and engineered follicle construction, while also advancing standardized manufacturing and consistent evaluation systems. In this process, precision medicine may help identify patients most likely to benefit from specific therapies, and tissue engineering may provide new sources for follicle reconstruction. As humanized models, biofabrication, and multi-omics approaches are integrated, the conditions for translating hair follicle regeneration technologies into clinical practice may gradually mature.

## Glossary

AA: alopecia areata
AGA: androgenetic alopecia
ASCs: adipose-derived stem cells
ATMPs: advanced therapy medicinal products
CGF: concentrated growth factor
DMNs: dissolving microneedles
DPCP: diphenylcyclopropenone
DP: dermal papilla
DPCs: dermal papilla cells
DSCCs: dermal sheath cup cells
ECM: extracellular matrix
EMI: epithelial–mesenchymal interaction
ESCs: epidermal stem cells
FUE: follicular unit excision
GMP: good manufacturing practice
HFGs: hair follicle germs
HFSCs: hair follicle stem cells
HG: hair germ
hiPSCs: human induced pluripotent stem cells
hUCMSCs: human umbilical cord mesenchymal stem cells
i-PRF: injectable platelet-rich fibrin
JAK: Janus kinase
MNRF: microneedle radiofrequency
NCs: nanocrystals
NLCs: nanostructured lipid carriers
OCEBM: Oxford Centre for Evidence-Based Medicine
ORS: outer root sheath
PEVs: platelet-derived extracellular vesicles
PL: platelet lysate
PLGA: poly(lactic-co-glycolic acid)
PRF: platelet-rich fibrin
PRP: platelet-rich plasma
RCTs: randomized controlled trials
SALT: Severity of Alopecia Tool
Shh: Sonic hedgehog
SVF: stromal vascular fraction
TACs: transit-amplifying cells
